# ‘How Does Nothing Show Up When I'm in So Much Agony?’: A Qualitative Study Exploring Patient Experiences of Non‐Specific Abdominal Pain in Acute Surgical Care

**DOI:** 10.1111/hex.70540

**Published:** 2025-12-31

**Authors:** Anna Kathryn Taylor, Daniel Romeu, Jess Dodd, Catherine Moriarty, Adam Peckham‐Cooper, Max Henderson, Elspeth Guthrie, Giles Toogood

**Affiliations:** ^1^ School of Medicine, Leeds Institute of Health Sciences, Faculty of Medicine and Health University of Leeds Leeds UK; ^2^ Lived Experience Co‐Researcher Leeds UK; ^3^ Department of General Surgery St James' Hospital, Leeds Teaching Hospitals NHS Foundation Trust Leeds UK

**Keywords:** abdominal pain, acute surgical care, patient experience, qualitative research, thematic analysis, unexplained symptoms

## Abstract

**Introduction:**

Non‐specific abdominal pain is a common and diagnostically challenging presentation in acute care, yet little is known about patient experiences within this setting. This study explores the experiences of patients attending a surgical same‐day emergency care (SDEC) unit with non‐specific abdominal pain.

**Design:**

Qualitative descriptive study using inductive thematic analysis.

**Methods:**

23 adults (aged ≥ 18 years) presenting with acute non‐specific abdominal pain to a surgical SDEC in England were purposively sampled. Semi‐structured interviews were conducted at least 2 weeks after discharge and thematically analysed inductively, iteratively and collaboratively by a team of psychiatrists, surgeons and a lived experience co‐researcher.

**Results:**

Three themes were identified: (1) **The journey to the SDEC**—participants described uncertainty and fear about potential diagnoses and varied thresholds for help‐seeking, (2) **The consultation**—while many appreciated rapid access to care, experiences of communication and explanation were mixed, with some feeling dismissed or confused by the absence of a clear diagnosis, and (3) **Post‐consultation reflections**—some felt reassured by normal test results, while others struggled with persistent symptoms, a lack of follow‐up, and ongoing uncertainty. Discussions around psychosocial factors were rare.

**Conclusions:**

Acute non‐specific abdominal pain can be distressing for patients, even after attending acute surgical services, particularly when communication is perceived to be unclear and follow‐up is inconsistent. A more structured, patient‐centred approach, including standardised follow‐up, clear explanations and sensitivity to psychosocial factors, could improve experiences and possibly outcomes for this group.

**Patient and Public Contribution:**

A patient and public involvement and engagement (PPIE) group, comprising individuals with lived and living experience of persistent physical symptoms, shaped the scope and design of the research and co‐produced the interview topic guide. A lived experience representative was actively involved in data analysis, interpretation and manuscript preparation.

## 1 Introduction

Acute abdominal pain is defined as pain of non‐traumatic origin with a maximum duration of 7 days [1] and is the most common presentation to surgery [2], as well as accounting for 4%–10% of all emergency department (ED) attendances [3]. Same‐day emergency care centres (SDECs) have been introduced to rapidly assess and investigate patients presenting to the hospital with acute abdominal pain, with the aim of reducing ED attendances, reducing hospital admissions, and improving the patient experience [4]. The most common diagnosis used is non‐specific abdominal pain, which is arrived at when all investigations have been normal, and a systematic review found that this diagnosis was reached in between 22% and 44% of cases [5].

Although many people present to healthcare services with symptoms that cannot be easily explained by identifiable pathological mechanisms [6], acute non‐specific abdominal pain has received less attention than chronic non‐specific abdominal pain. Chronic non‐specific abdominal pain is regarded as a disorder of brain–gut interaction, which involves aberrant functioning of a variety of interlinked mechanisms, including intestinal sensorimotor function, mucosal and immune activity, gut microbiota and central nervous system processing [7]. The condition is associated with a high prevalence of psychological comorbidity (40%), which can both precede the onset of abdominal pain and be a reaction to it [8].

It is unclear whether the mechanisms which underlie the development of chronic abdominal pain are similar to those for acute non‐specific abdominal pain. A recent study suggests patients who present with acute abdominal pain have high rates of psychological symptoms, irrespective of whether the pain is explained or non‐specific [9]. Earlier work suggested patients who presented with acute abdominal pain and underwent appendectomy were more likely to report adverse life events prior to the onset of the pain if their appendix was histologically normal as opposed to inflamed [10]. Other research has found that patients with non‐specific acute abdominal pain have a poorer long‐term outcome than patients with comparable organic disease, such as acute appendicitis [2]. Almost three times as many patients with non‐specific abdominal pain (30%) compared to those with organic disease (11%) may go on to develop chronic non‐specific abdominal pain over the next two decades [2].

In the absence of clear organic pathology and uncertainty about potential underlying pathophysiological mechanisms, providing an explanation for patients' symptoms can represent a challenge for clinicians. Maatz et al. found that the word ‘difficult’ was commonly used by secondary care clinicians from medical and surgical specialties to describe the experience of diagnosing, explaining, communicating, and managing non‐specific symptoms [11]. In the acute surgical setting, with limited consultation time and wider service pressures, effective communication may be more challenging. Indeed, a systematic review found that surgeons frequently do not explore patients' emotions or concerns [12]. Clinicians report little or no formal training in how to manage individuals with non‐specific symptoms and therefore use variable explanations and strategies to communicate this concept [13], highlighting the potential need for a more informed and unified approach.

Patients who feel they have not had a clear explanation for their symptoms commonly report the need to feel understood and validated, and they search for an explanation that makes sense to them [14]. It can be difficult for patients to face uncertainty about their symptoms, and patients often express disappointment in the healthcare system due to perceptions of being dismissed and uncared for [14]. Understanding the specific perspectives of patients with non‐specific symptoms in an acute surgical setting is essential to improving patient‐centred care and optimising outcomes for these individuals.

The aim of this study was to understand the experiences of care among individuals presenting to an emergency surgical setting with acute non‐specific abdominal pain, and their understanding of the nature of their pain following discharge.

## 2 Methods

This study used qualitative descriptive methodology [15]. Semi‐structured qualitative interviews were conducted with patients following their visit to a surgical SDEC in a large teaching hospital in England. This study represented one component of a larger research project exploring the presentations and outcomes of people who, following assessment and investigation, received a diagnosis of acute non‐specific abdominal pain; the full protocol of the multicomponent study is described by Romeu et al. 2023 [16]. Ethical approval for this study was granted by the Hampstead Research Ethics Committee via the Health Research Authority (REC reference 22/LO/0734, IRAS ID 314748). This exploratory qualitative study is reported following the COnsolidated criteria for REporting Qualitative research (checklist) by Tong et al. (2007) [17].

### 2.1 Recruitment

**Figure 1:**
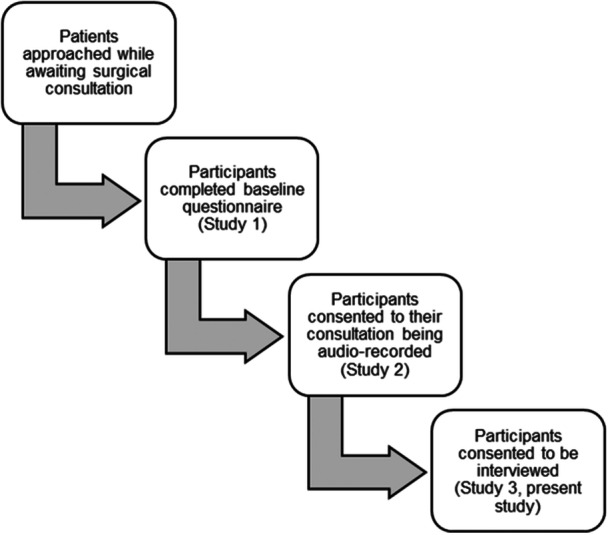
Flow diagram demonstrating participant recruitment for the present study within the context of the wider multicomponent study.

Participants were eligible for inclusion if they had participated in the preceding parts of the study. The study (see Figure 1) involved adult (aged ≥ 18 years) participants who presented with acute abdominal pain to a surgical SDEC unit. While in SDEC, they completed a questionnaire about gastrointestinal symptoms, quality of life, anxiety and depression. Their diagnostic consultation was then recorded. Purposive sampling [18] was then used to identify a diverse sample of participants for this qualitative study, which focuses specifically on those participants whose symptoms were identified by clinicians as being non‐specific in nature. The researchers aimed to gain a sample which was diverse in terms of gender, age, ethnicity and education levels in order to include underserved groups, such as ethnic minority populations, who are frequently under‐represented in research studies.

Eligible participants were contacted by a member of the research team via telephone to gauge interest in participation. Following expressions of interest, they were sent a participant information sheet. Participants were advised that they could withdraw their consent at any time until the dissemination of results. Written consent to participate and for interviews to be audio‐recorded was obtained prior to conducting interviews.

### 2.2 Data Collection

Between March and November 2024, 23 individual semi‐structured interviews were conducted by researchers with qualitative methods expertise (A.K.T. and D.R.) who also made field notes. All interviews were conducted via telephone or Microsoft Teams to maximise accessibility and flexibility for participants, and audio‐recorded. Telephone and online consultations have been implemented successfully within both acute and university settings, including for research on sensitive and potentially traumatic topics [19]. Using a semi‐structured interview approach ensured that all relevant topics were covered, while also enabling participants to talk about other areas that they also felt were important [20, 21]. Interviews were conducted at least 2 weeks after their initial presentation to the surgical unit. Participants were asked to participate in the interview from a private space to encourage their open and honest reflection; likewise, interviewers were alone at the time of the interview.

A topic guide was drafted from the existing literature and developed iteratively within the research team and with input from a patient and public involvement and engagement (PPIE) group. Interviews explored participants' experience of admission to the SDEC for their abdominal pain, the course or persistence of the pain since their discharge, their recollection and understanding of the clinician's explanation, further help‐seeking behaviours, and any current physical or psychological difficulties. The semi‐structured interview guide is included as an appendix. All participants were offered a £20 shopping voucher as a token of appreciation for their participation.

### 2.3 Data Analysis

Demographic data, including age, gender, ethnicity and education level, were collected from the questionnaire that participants completed on entry to the wider research study. Interview recordings were transcribed verbatim, anonymised and checked for accuracy before being permanently destroyed. The analysis team included four psychiatrists (A.K.T., D.R., E.G. and M.H.), two general surgeons (A.P.C. and G.T.) and a lived experience representative (J.D.).

The six steps as outlined by Braun and Clarke [22] were followed: familiarisation with the data, generalising initial codes, searching for themes, reviewing themes, defining and naming themes, and producing the report. These steps were operationalised as outlined. Interview transcripts were manually coded line by line and analysed inductively using thematic analysis [22]. Following data familiarisation, AKT and DR independently analysed the interviews and assigned codes to participant descriptions of their experiences of presenting to the ambulatory surgical centre with abdominal pain, receiving the outcome of non‐specific abdominal pain, and their thoughts and feelings in the following weeks. A subset of transcripts was reviewed independently by J.D. These codes were reviewed, and three main themes were identified to organise these experiences in chronological order. The codes were then discussed collaboratively within the wider research team, who collectively agreed on key themes and important sub‐themes. As analysis progressed, the thematic framework was continually refined using the principles of constant comparison, enabling adjustment of themes and codes [23, 24, 25]. The final themes were agreed upon by all team members.

Recruitment was paused after 20 interviews to undertake preliminary analysis and to review progress towards data saturation [26]. A further three participants were recruited and interviewed; no new themes or sub‐themes were identified during these interviews, thus recruitment was stopped as the researchers agreed that data saturation had been reached.

### 2.4 Patient and Public Involvement Statement

A PPIE group, comprising individuals with lived and living experience of persistent physical symptoms, was established during study conceptualisation. The group shaped the scope and design of the research and co‐produced the interview topic guide. In addition, a lived experience representative (J.D.) was actively involved in data analysis and interpretation and manuscript preparation. She independently reviewed a diverse subset of interview transcripts and identified key themes and reflections. Collaborative discussions facilitated the incorporation of these themes into the final framework. She reviewed, edited and approved the final manuscript.

## 3 Results

Individual interviews were conducted with 23 participants. Interviews were between 11.48 and 39.18 min in duration (mean 24.04 min). Participants were 78% female (*n* = 18) and 91% white (*n* = 21), with a variety of ages and levels of education. Participant demographic characteristics are summarised in Table 1. Some participants presented with abdominal pain alone, while others experienced associated symptoms including back pain, nausea, vomiting, abdominal bloating, changes in bowel habits and fatigue.

**Table 1:** Participant demographic characteristics.

	*N* (%)
Gender (female)	18 (78.2)
Ethnicity (white)	21 (91.3)
Age (years)	
18–25	2 (8.7)
26–35	7 (30.4)
36–45	7 (30.4)
46–55	1 (4.3)
56–65	3 (13.0)
66 and over	3 (13.0)
Education	
None	1 (4.3)
O Level/GCSE/NVQ2 or equivalent	2 (8.7)
A Level/NVQ3 or equivalent	6 (26.1)
NVQ4 or equivalent	1 (4.3)
Bachelor's degree or equivalent	6 (26.1)
Postgrad certificate, diploma, Master's or equivalent	6 (26.1)
Prefer not to say	1 (4.3)

The following themes will be presented in this paper, with illustrative quotations identified by a pseudonym for each participant: (1) the journey to the SDEC, (2) the consultation and (3) post‐consultation reflections.

1. ‘**This Isn't Right, There's Something Wrong’: The Journey to the SDEC**
  Most participants had experienced abdominal pain for several days before seeking help from medical professionals.
  It was a gradual onset kind of pain, but then suddenly got worse as the day went on…. But then after a week, the pain wasn't getting any better.
  Sade
  Some had longer‐term symptoms that intensified over several weeks before seeking medical attention. Participants with experience of chronic pain reflected that this made it difficult to know how and when to seek help.
  I deal with pain on a daily basis, but it was too much for me to kind of handle, and it was really abnormal, the amount of pain that I was having.
  Sarah
  Participants described a number of concerns that they had had about the possible cause of their symptoms. The most common worries were appendicitis and gallbladder problems.
  I was really confused because like at first I was like, I think I've got appendicitis because, like, all the symptoms showed up apart from, like, the vomiting and stuff.
  Chloe
  A minority of participants did not have any specific concerns about the cause of their symptoms before seeking medical attention.
  I didn't have a clue…. I'm not the sort of person to, like, get worried, or, you know, you get told what it is, you can't, you can't worry about it, can you?
  Nick
  In contrast, some participants reported that they had been worried about a more significant diagnosis, such as cancer; for some, this was linked to family members or personal contacts who had had these conditions. These participants may require additional reassurance.
  I think as well, laying awake at night when you're in pain and then thinking you've got cancer, who's going to look after everything, who's going to pay the bills?
  Jessica
  My immediate thoughts were it's my gallbladder…. And I have had acquaintances who have had gallbladder pain, and my father also actually died from gallbladder infection.
  Nancy
  Almost all participants initially sought advice from their General Practitioner (GP), who either arranged an appointment with the SDEC directly or signposted them to the ED, where ED clinicians referred them onward to the SDEC.
  When the GP pressed on, sort of the right hand side, it was quite, it was quite severe. It was more the, you know, the pressure, it was really severe. And they were just a bit concerned that could be my gallbladder, I think? So they sent me off to the ambulatory clinic.
  Sandra
  This was usually recommended as a way of quickly excluding dangerous or serious pathology.
  He [doctor at urgent centre] was like, if it is appendicitis, I can't risk you going home because obviously it can rupture any time. So, then he was like, I'm sending you to A&E.
  Chloe
2. ‘**When Nothing Shows up, There's Not Much They Can Do’: The Consultation**
  Participants generally spoke positively of the SDEC as an alternative to ED. They valued being able to receive investigations and discuss their concerns with clinicians more quickly.
  I wasn't there for very … a couple of hours, maybe three hours? And then I came back the next day for an ultrasound, so I found the whole thing quite a pleasant experience, to be fair, compared to previous times when I've had to go through A&E and wait and go back and different consultant appointments and one thing and another. Whereas I found it really, really, the whole thing from start to finish, kind of, being seen, having my bloods and then speaking to the doctor all in the same day, it were really good.
  Elizabeth
  All participants recalled having blood tests; most also recalled having an ultrasound scan either on the day of presenting to the SDEC or in the days following, organised following an initial consultation and with the results discussed in the SDEC clinic.
  I'd just sat down and I was whipped off to see the radiologist who gave me the ultrasound.
  Carla
  Participants' experiences of their diagnostic consultations after undergoing investigations were variable. Some spoke positively, explaining that they thought the assessment was thorough and addressed their concerns.
  He was very thorough. He was good. Put me at ease. As soon as I knew there wasn't anything, you know, seriously, seriously wrong with me then you feel more at ease.
  Annette
  Others were disappointed, feeling that the consultation had not adequately explored and responded to their concerns. They felt that their symptoms and worries had not been listened to, and they were confused and distressed by the fact that there was no explanation they could understand.
  I didn't actually feel like anybody was listening to me…. And I felt completely lost and very much alone. Because I felt that the people who are supposed to help you get better just weren't sort of listening to what I was saying, if that makes sense…. He asked me to go through the symptoms and what brought me there again, he examined me. He just said “we've got nothing else to add.” I just couldn't believe that there's no answer.
  Angela
  Many participants felt that they could not understand the decision to discharge them from the SDEC without a clear diagnosis or explanation for their symptoms, even if the clinician had suggested a potential cause or causes. They felt that although investigations had been normal, because there was a perceived lack of definitive diagnosis they should be offered further investigations or medication beyond pain control.
  They said maybe it were fatty liver that were contributing towards the uncomfortableness. But then kind of just said “you need to go back to your GP.” I kind of asked if it could be anything else, and it were a bit like “we've done our bit now, you need to go back.” Which I suppose if the pain had got worse, I'd have felt a little bit kind of…. That I'd ended up back where I started if that makes sense?
  Elizabeth
  He told me my results show that there's nothing wrong with me, because they couldn't find anything. They did a scan and everything. Everything came out well, so…. They just couldn't figure it out. They said it's a virus or something. Which I believe there's more because I've been through this pain several times. So I was suggesting to him, can you at least give me something like omeprazole or something, just to ease my pain. But he said no, I should just go for painkillers, but it was not working.
  Blessing
  Some described feeling dismissed, angry or like a ‘fraud’ for attending the hospital with symptoms that could not be explained by medical tests, particularly if they did not feel there had been a clear plan offered by the clinician. However, some participants also acknowledged that it was possible for clinicians to not reach a definitive diagnosis.
  When nothing shows up, there's not much that they can do, especially since like they've done a scan and you can't see anything there, like I don't know, like I think back then I was a bit annoyed because I was like, how can nothing show up, show up and how can like, you know, how can I be in this much agony and like, I feel like they're not really doing anything. But … they couldn't have done much else at that moment because they don't know. Like it's unexplainable at that moment in time.
  Chloe
  Only one participant reported that stress or mental health had been discussed as a potential explanation for his symptoms, which he was able to accept as a reasonable possibility.
  I was sort of really stressed…. Neither of us really knew what was sort of going on and it's always a little bit of guesswork, but everything that I told them it seemed like they had taken it into consideration and…. That's what they'd came up with, and it all made sense. I never left thinking “this feels like something else” if you know what I mean…. They'd done the scans, they'd sort of listened to what I'd been going through, what had happened. It seemed like they came to a good, sort of educated conclusion…
  Danny
  Participants had varying experiences of follow‐up care and advice. A minority recalled being given information about patient‐initiated follow‐up, which would enable them to return to the SDEC within a certain time period if their symptoms did not resolve. A few had further investigations organised, or were referred to a different specialty, such as gynaecology.
  [The consultant] basically said, if this happens again, just you know come again, and I said, wait, I don't want to wait 12 hours in A&E again to see you, and he, he was very, erm, very good, he just said no, if it happens just come here directly…. I will see you, so that was for me amazing, you know, I felt like I have someone to call if this happens again, I won't start the whole cycle again, waiting in A&E…
  Mark
  I think he said it'd be unusual for the spot on my liver to be causing the pain. But I think he probably just felt like just to be on the safe side, just to rule everything out, just in case because there is…. I remember he showed me kind of the spot on the scan. And he said just to be on the safe side, to rule it out, we'll kind of do that and I don't know if he also thought, maybe we'll see if anything else pops up potentially…. But I think it felt more like him just kind of going just to, yeah, check all the boxes to say that we've definitely checked everything.
  Stephanie
  Others described being offered analgesia, including opiates, but struggled to accept escalating regimes of analgesia without a clear understanding of the cause of their pain.
  He prescribed me with some codeine for pain…. He sort of literally just said that it was just abdominal pains and to have painkillers…. Wasn't very helpful in concerns of like, what I should do if anything else happens or anything like that.
  Sophie
  Many were discharged to the care of their GP with no further follow‐up or advice. Participants reported finding this difficult to understand as they were unsure what their GP would be able to offer to help them understand or manage their pain.
  I don't think I was signposted to anywhere else. It was kind of just like “we've not found anything. You need to go back to the GP.” That was sort of it.
  Emily
3. ‘**I Had to Bring My Pain Home and Struggle’: Post‐Consultation Reflections**

For many participants, their pain symptoms resolved with time, though some continued to experience persistent symptoms.

I was sort of more relieved than anything. The fact that I could physically feel this pain going away, that my movements, lateral movements were becoming easier…. So, as it started to abate, I just, I just recall feeling relieved that there wasn't anything seriously wrong.

Charles

So at the moment I'm sort of…. I don't know, maybe it isn't my gallbladder, but I don't know, that's the problem. I'm in that sort of situation of, I don't really know.

Stephanie

Some participants, including a few with persistent symptoms, reported feeling relieved that the investigations undertaken at SDEC had shown no abnormality and that this had given them some peace of mind.

A little frustrated that if it isn't something they can treat, it might come back and everything. But you know what? They can't do anything about that, can they? But on the whole…. I was just relieved to be there and have the tests and know that everything was sort of OK.

Sandra

A few participants had their own ideas about what had caused their symptoms, despite investigations finding there was no clear evidence of this. However, they found this explanation and their own self‐management strategies reassuring.

As soon as I cut out gluten and started buying gluten free products, I started to feel quite a bit better…. But you've got to put things to age as well…. I've had a turnaround since I changed my diet and lost a few pounds and I'm just thinking “right, deal with it girl”…. Yeah, you can't just take tablets for everything, it's not always about tablets.

Annette

However, the remaining participants struggled with the fact that their symptoms remained non‐specific, particularly for those whose pain had persisted. These participants wished for more investigations and assessment, fearing that something significant had been missed that might lead to further illness or even death.

I think I was frustrated that nothing showed up that, yet again I'm having something, had something done that didn't kind of explain why I'm feeling this pain, um, I, I was sad, I mean also happy, but it's, I mean, I, people often say that when obviously things come back fine, you're like “hoorah, I'm fine” but I know that I'm not…. I do not kind of feel like I was palmed off or anything, but it's just frustrating for me because I'm still, kind of, don't have any answers.

Sarah

They struggled to tolerate the uncertainty of not having a clear explanation for their symptoms, despite having multiple investigations, and expressed anxiety around not knowing how to get answers now that all the tests had been normal. Participants reflected that although they could, to a degree, accept that there was not an answer, they also feared that there might be something more sinister that had been missed.

Kind of processing all the tests and things, actually coming to terms of the fact that sometimes there just isn't an answer. But also putting my faith in the fact that these scans and all the rest of it, haven't shown anything significant. I think that was my main worry that something underlying had been missed. But I suppose with all the blood tests and the MRI scan and the other scan…. I've just kind of got to put my faith in the system, but then you hear stories that things have been missed and then, you know, six months down the line, people have discovered not very pleasant things. So that's always in the back of my mind.

Angela

Only a few of those with persistent symptoms had sought further help from general practice, leaving them unsure of who they should seek help from and a perceived lack of options.

I'm reluctant [to go to the GP] because it's always the same answer that you get, you don't get a positive answer, you don't get results.

Blessing

However, the majority of participants did not return to their GP because their symptoms had resolved on their own and, as a result, felt that they did not need further support from general practice.

## 4 Discussion

### 4.1 Summary of Findings

We believe that this is the first qualitative study to explore the experiences of patients attending a surgical SDEC unit with symptoms later classified as non‐specific abdominal pain. Findings highlight the benefits and challenges of seeking acute surgical care for these presentations. Participants valued rapid access to investigations and surgical reviews outside of the ED, but their subsequent experiences diverged. While some were reassured when no pathology was identified and their pain resolved, others were unsettled by persisting symptoms, worried that a diagnosis had been missed and frustrated by a lack of clear explanation or follow‐up.

There appeared to be a lack of consistency in who was informed of patient‐initiated follow‐up, and participants at times struggled to accept that they could experience pain without a clear cause. The majority of participants' pain resolved with time, and they were reassured by the lack of pathology identified by the investigations at SDEC. Although only a minority experienced persistent pain, they reported being deeply impacted by it, expressing worry that a sinister cause had been missed.

### 4.2 Strengths and Limitations

Strengths of this study include embedding PPIE throughout design and analysis to maximise acceptability and relevance to those with lived experience of unexplained pain. A further strength was a multidisciplinary research team incorporating surgeons, psychiatrists and people with lived experience of non‐specific abdominal pain, which enabled richer interpretation of the data [27]. Participants were aware that interviewers (A.K.T. and D.R.) were independent from their clinical care, which may have facilitated candour. The use of semi‐structured interviews enabled participants to speak openly about the experiences of help‐seeking they had found important or particularly meaningful to them, while still ensuring that the areas detailed in the topic guide were covered. Interviews were conducted either via telephone or Microsoft Teams according to participant preferences, which improved accessibility, reduced digital exclusion and encouraged participation from a range of backgrounds.

There were some limitations of the study. Although the interviews were, when possible, conducted within 2–4 weeks of the participants' attendance at SDEC, there is the potential for recall bias. As with many qualitative studies, interview participants may also have been more likely to participate if they had stronger views (positive or negative) associated with their SDEC attendance. Although those contacted were interested in participating, it was often difficult to reach potential participants to invite them to join the study during normal working hours, which may bias the sample towards participants who were able to answer the phone during the day or work more flexibly. However, once contact was made, only one potential participant did not attend the interview; all other people who had agreed did attend the interview. The sample was predominantly white and female, albeit there was a range of ages and educational levels. Given the urban setting of the recruitment site, the findings of this single‐centre qualitative study might not be transferable to other geographical areas.

### 4.3 Comparison With Previous Literature

We identified mixed participant experiences in our study. Participants were typically either reassured by investigations and felt relieved, particularly when the pain resolved on its own, or worried by a lack of clarity in the diagnosis of non‐specific pain. Sowińska et al. (2018) reported that patients with non‐specific or persistent physical symptoms may develop their own frameworks to explain the origin of these symptoms, which can include external factors such as stress, or internal factors including both psychological and physical factors [28]. Although our participants had had acute symptoms rather than long‐term pain, our results found that some had started to develop their own ideas for what had caused these symptoms, such as gluten insensitivity or stress.

A small number of participants had experienced non‐specific symptoms before, and these patients appear to face additional barriers. Differentiating new symptoms from baseline discomfort can be difficult, and prior negative experiences of not feeling believed or listened to may undermine trust and influence their perception of future consultations [29]. Tailored advice on pain management at home, sensitive to the patient's context and preferences, could address some of these gaps.

A previous study found that hospital clinicians find it difficult to manage non‐specific symptoms due to a lack of training and experience, with further investigations being ordered without a clear rationale and variable explanations of normal results [13]. Our study also identified that some patients were referred for further investigations or had been recommended stronger analgesia without what the patient perceived as an understanding of its indication. Surgeons working in an acute setting with limited prior knowledge of the patient and limited time may find managing non‐specific pain particularly challenging and may not feel they have the time, resources or expertise to explore psychosocial factors [30, 31].

Patients and clinicians may have different ideas about what a ‘good outcome’ of the consultation means. While patient participation and establishment of a therapeutic relationship are core components of person‐centred care, there can be variation in the degree to which this is done depending on the professional group and setting [32]. Managing non‐specific symptoms in the acute surgical setting is uniquely challenging. High patient turnover, as well as competing demands and time pressures, may limit opportunities for detailed exploration of patient concerns. Clinicians may view a consultation as successful if serious pathology has been excluded; however, patients may experience ongoing uncertainty if their pain remains unresolved, which can lead to disappointment in health services rather than reassurance [14]. This may be exacerbated by a lack of a clear follow‐up mechanism. The potential implications of patients not feeling they have had an explanation of their symptoms are that one‐third may develop chronic abdominal pain [2] or may attend primary or secondary care frequently, looking for a satisfactory answer [33]. This can contribute to the high economic burden of non‐specific symptoms, particularly when they are persistent [33].

### 4.4 Implications for Research and Practice

Our study identified that communication was central to patient experience. Participants expressed a clear desire for an explanation of their symptoms, and presentation of normal test results accompanied by reassurance from doctors has little impact on patients' doubts or anxieties. Effective reassurance required tangible explanations that participants could understand, and there is existing guidance for clinicians on how to explain chronic non‐specific pain [34, 35]. An example explanation is that the brain and the gut communicate with each other through nerves and chemical signals, which are outside conscious awareness most of the time. However, normal brain–gut communication can be disturbed by physical factors such as infection or psychological factors such as stress, and when that happens, the brain may perceive the gut's signals more strongly or may send inappropriate signals to the gut that then disturb its functioning [35]. Although this model is for chronic pain, future research could adapt it to an acute surgical setting. In addition to giving patients an explanation for their symptoms, this approach may also increase clinicians' confidence in managing non‐specific symptoms.

Giving greater consideration to the role of a wider MDT may also be valuable to the care of this patient group. A previous review found that an approach incorporating physical, pharmacological and psychological interventions was most effective in managing patients with persistent physical symptoms in primary care, and adapting aspects of such interventions and the healthcare professional delivering them may offer meaningful care to patients in the acute surgical setting [36].

Psychosocial factors also warrant careful consideration. It is not always necessary to attribute non‐specific pain to psychological causes [37]. Clinicians need confidence to identify and sensitively respond to psychosocial cues, avoiding unnecessary investigations while not prematurely attributing symptoms to psychological causes. Building such nuance into acute encounters can be challenging, but may reduce iatrogenic harm and foster trust.

Our study found a lack of standardised follow‐up. Offers of patient‐initiated review varied by clinician, leaving some patients unsure of how to seek help if symptoms persisted or worsened. Reliance on patients to organise general practice‐based follow‐up placed an additional burden during a time of uncertainty. Patient‐initiated follow‐up is commonly used in health services [38], and adapting the SDEC discharge process to include a clearer follow‐up mechanism may reduce anxiety and support continuity. Patient‐initiated follow‐up could be offered as an in‐person appointment, or through a telephone call to establish whether an in‐person review is needed to maximise efficiency. Additionally, a leaflet could be developed to give to patients to take with them upon discharge, giving them clear information about how to use the patient‐initiated follow‐up system and giving information on how to manage symptoms at home.

## 5 Conclusions

Patients presenting with non‐specific abdominal pain to acute surgical settings describe mixed experiences, influenced by the perceived adequacy of explanations and clarity of follow‐up. Most people reported feeling reassured by normal investigation results, but a significant minority continued to experience distress and uncertainty, with the potential for persistent pain and repeated healthcare use. Improving patient experiences in this setting may be achievable through simple interventions, including clear explanations about investigation findings and possible mechanisms of pain (including non‐specific pain) using accessible explanatory models. Introducing a standardised discharge and follow‐up process, potentially incorporating an information leaflet with guidance on patient‐initiated follow‐up and self‐management advice, may improve the experiences of all patients with non‐specific pain and potentially reduce the likelihood of patients developing persistent symptoms. Future research should test the feasibility and effectiveness of these approaches in SDEC and other acute care settings and explore their impact on patient outcomes, healthcare use and clinician confidence in managing non‐specific presentations.

## Author Contributions

**Anna Kathryn Taylor:** conceptualisation, study design, funding acquisition, ethical approval, data collection, initial analysis, analysis refinement, writing – original draft, writing – review and editing, project administration. **Daniel Romeu:** conceptualisation, study design, funding acquisition, ethical approval, data collection, initial analysis, analysis refinement, writing – original draft, writing – review and editing, project administration. **Jess Dodd:** initial analysis, analysis refinement, writing – review and editing. **Catherine Moriarty:** participant recruitment, analysis refinement, writing – review and editing, project administration. **Adam Peckham‐Cooper:** conceptualisation, study design, analysis refinement, writing – review and editing, supervision. **Max Henderson:** conceptualisation, study design, analysis refinement, writing – review and editing, supervision. **Elspeth Guthrie:** conceptualisation, study design, analysis refinement, writing – review and editing, supervision. **Giles Toogood:** conceptualisation, study design, analysis refinement, writing – review and editing, supervision.

## Ethics Statement

Ethical approval for this study was granted by the Hampstead Research Ethics Committee via the Health Research Authority (REC reference 22/LO/0734, IRAS ID 314748).

## Consent

Written informed consent for publication of anonymous quotations was obtained from all participants prior to data collection.

## Conflicts of Interest

A.K.T. is a member of the ECR Editorial Board of Health Expectations. The other authors declare no conflicts of interest.

## Supporting information

COREQ Checklist.

Topic guide.

## Data Availability

Due to the sensitive nature of the data and the potential for participant identification, the dataset is not publicly available. Data are available from the corresponding author upon reasonable request.
