# Supplementary material for: ‘How Does Nothing Show Up When I'm in So Much Agony?’: A Qualitative Study Exploring Patient Experiences of Non‐Specific Abdominal Pain in Acute Surgical Care
Source: Health Expect. 2025 Dec 31;29(1):e70540. doi: 10.1111/hex.70540 (PMC12754566; doi:10.1111/hex.70540)
Supplement: Supplementary file 2 — Topic guide. [file HEX-29-e70540-s002.docx]

**INTERVIEW TOPIC GUIDE**

**Presentations and outcomes of people with unexplained symptoms in Acute General Surgery: a mixed-methods study**

Introductions and confidentiality

Introduce self and role.

“Thank you for speaking with me, and for your participation in the study so far. Today I am interested in hearing about the pain that brought you to hospital, and your experiences of receiving care on the Ambulatory Surgical Centre. I would also like to hear about your consultation with the surgeon, and how you understand your symptoms now.

There are no wrong or right answers, and nothing you say will affect any clinical care you will receive in the future. What you say will be confidential, except if you express any intent of harming yourself. If that happens, I may need to break confidentiality to keep you safe. If at any point you wish to stop the interview or you become upset, we can stop. We can then take a break and carry on or stop altogether. If you do not want to answer any specific questions, please say so, and we can skip it and move on. Are you happy to continue?”

Consent and recording

Go over consent form and obtain signatures.

“Is it alright if I record our conversation? You are welcome to request a copy of the transcript for you to read if you would like. To protect your privacy, I will not be using your name during the interview. Once I start recording, I will state your study ID number and ask for permission to continue. Remember that you can ask to pause or break at any time, and please let me know if any of the questions are unclear. Are you ready to begin?”

Topic guide

*The italicised questions will only be used as prompts if the initial open question elicits little narrative.*

1. Tell me about the pain that brought you to hospital.
   - *How did the pain start? When did it start?*
   - *Did you seek help from elsewhere before coming to hospital?*
   - *Were you referred by your GP or did you come to hospital yourself?*
   - *What did you think was the cause of your pain?*
   - *Did you experience any other symptoms?*
2. Tell me about your experience of receiving care on the Ambulatory Surgical Centre.
   - *What was positive about the experience?*
   - *What could have been improved?*
   - *Did you have to wait a long time to be seen?*
   - *Did you feel that your concerns were taken seriously?*
   - *Did the staff do their best to help you and to make you feel comfortable?*
   - *How did the experience compare to your expectations?*
3. What can you tell me about your consultation with the surgeon?
   - *How did the surgeon explain the cause of your symptoms?*
   - *Did the explanation make sense to you?*
   - *Was the explanation clear?*
   - *Did the surgeon address your concerns?*
   - *How did you feel at the time?*
   - *Did you feel that the surgeon spent enough time with you?*
   - *Did you receive any written information? Were you signposted to any resources?*
4. Do you think the explanation of your symptoms could have been improved?
   - *How could it have been improved?*
   - *What else would have been helpful?*
5. How have your symptoms been since you were discharged from hospital?
   - *Are you still experiencing abdominal pain? Has it changed at all?*
   - *Do you think you are better off having visited the Ambulatory Surgical Centre?*
   - *Is the pain affecting your life or your ability to cope?*
   - *Have you sought medical attention since your visit to hospital?*
6. How do you understand your symptoms now?
   - *What do you believe is the cause of your abdominal pain?*
   - *Which factors do you think are contributing to your pain?*
   - *Do you think anything else might be contributing to your pain?*
7. Are you experiencing any other difficulties?
   - *How have you been feeling in general?*
   - *How would you describe your mood?*
   - *Are you worried about anything?*
   - *Do you have any other physical symptoms that are bothering you?*
   - *How do these problems affect you?*
8. Are there any other ways that the team could have improved the care and support provided to you?

**I have no other questions; is there anything else you would like to add about your experience that I have not asked about?**

*Closing statement:*

“On behalf of the whole research team, thank you very much for participating in this study and sharing your experiences with me.”
